# Degradation of Wheat Germ Agglutinin during Sourdough Fermentation

**DOI:** 10.3390/foods10020340

**Published:** 2021-02-05

**Authors:** Luis E. Rojas Tovar, Michael G. Gänzle

**Affiliations:** Department of Agricultural, Food and Nutritional Science, University of Alberta, Edmonton, AB T6G 2P5, Canada; rojastov@ualberta.ca

**Keywords:** sourdough, proteolysis, fermentation, wheat germ agglutinin, wheat sensitivity, lactic acid bacteria

## Abstract

Non Celiac Wheat Sensitivity (NCWS) is an intolerance to wheat products and individuals with NCWS often adhere to a gluten free diet. However, gluten free diets are often associated with a reduced sensory and nutritional quality. Wheat Germ Agglutinin (WGA) is one of the wheat components linked to NCWS. This study explored the fate of WGA during sourdough fermentation. To assess the role of thiol-exchange reactions and proteolysis, sourdoughs were fermented with *Fructilactobacillus sanfranciscensis* DSM20451, *F. sanfranciscensis* DSM20451Δ*gshR*, which lacks glutathione reductase activity, or *Latilactobacillus sakei* TMW1.22, with or without addition of fungal protease. The conversion of WGA was determined by size exclusion chromatography of fluorescence-labeled WGA, and by enzyme-linked immunosorbent assay (ELISA). Commercial whole wheat flour contained 6.6 ± 0.7 μg WGA/g. After fermentation with *L. sakei* TMW1.22 and *F. sanfranciscensis* DSM20451, the WGA content was reduced (*p* < 0.05) to 2.7 ± 0.4 and 4.3 ± 0.3 μg WGA/g, respectively, while the WGA content remained unchanged in chemically acidified controls or in doughs fermented with *F. sanfranciscensis* DSM20451Δ*gshR*. Protease addition did not affect the WGA content. In conclusion, the fate of WGA during sourdough fermentation relates to thiol-exchange reactions but not to proteolytic degradation.

## 1. Introduction

Bread has been consumed by humans for 14,400 years or more [1] and wheat is most commonly used for bread production. However, wheat has been related to several health disorders [2]. Gluten proteins from wheat, rye and barley trigger celiac disease, an illness with a prevalence of about 1% of consumers that causes intestinal inflammation and reduces nutrient absorption in the intestine [3]. A more frequent disorder linked to wheat is referred to as non-celiac wheat sensitivity (NCWS). Diagnosis of NCWS is not based on specific biomarkers but on ruling out celiac disease and wheat allergies [4]. Wheat components related to NCWS are fermentable oligo-, di-, and monosaccharides and polyols (FODMAPs), amylase-trypsin inhibitors (ATIs), and wheat germ agglutinins (WGA) [4,5,6,7]. NCWS, which is also referred to as gluten sensitivity or gluten intolerance, involves a wide variety of symptoms, including bloating, diarrhea, nausea, and intestinal damage. Extra intestinal symptoms have also been described and may include tiredness, headaches, joint pain and anxiety [5]. Symptoms of NCWS overlap with irritable bowel syndrome (IBS) [6]. Individuals affected by NCWS are normally prescribed a diet which is free of wheat products [8].

Sourdough fermentation does not eliminate gluten proteins that trigger celiac disease but the use of sourdough processes in bread making can be an alternative to gluten free diets to reduce symptoms associated with NCWS. The sourdough process involves longer fermentation times in comparison to straight dough processes, and additionally recruits the metabolic activity of lactic acid bacteria. Sourdough fermentations partially or completely degrade FODMAPs in wheat [9,10] and provide more time and more suitable conditions for wheat aspartic proteases, which are optimally active at low pH, to degrade wheat proteins [11,12]. Wheat flour contains the serine carboxypeptidase D (*CPW-II*), an exopeptidase with an optimum pH from 4 to 5.5 [13]. The most important proteases in wheat are aspartic proteinases [14] that associate with gluten during mixing and are optimally active at acidic pH [15,16]. Wheat aspartic proteases hydrolyze peptide bonds adjacent to arginine, lysine, phenylalanine, leucine, tyrosine and tryptophan [15,17]. The pH was reported to be primarily responsible for sourdough effects on the structure and bioactivity of the wheat amylase-trypsin inhibitor (ATI) [18]. Moreover, sourdough lactic acid bacteria reduce disulfide bonds and decrease the redox potential of the dough. Specifically, glutathione reductase of *Fructilactobacillus sanfranciscensis* reduces oxidized, dimeric glutathione to glutathione, which further reacts to disrupt disulfide bonds in proteins, alters their secondary structure and promotes proteolysis of disulfide-bonded proteins [12,19,20].

WGA is a lectin that is located in the germ of the wheat grain. In the pH-range of 3.5 to 7.4, it forms a dimer with a size of approximately 35 kDa that is relatively heat stable [21,22,23]. Each monomer is stabilized by 16 intramolecular disulfide bonds [24]. WGA binds N-acetyl glucosamine and its β-(1→4)–linked oligomers and has weaker affinity to N-acetyl galactosamine and N-acetyl neuraminic acid [25,26]. WGA’s effects on human health are controversial. Rodent experiments concluded that WGA in doses that substantially exceeded the concentration in wheat decreased growth [27]. In cell culture experiments with Caco2 cells, WGA increased the permeability of the epithelial layer [28] and stimulated synthesis of pro-inflammatory cytokines [29]; WGA also demonstrated toxicity to acute myeloid leukemia cells without significant toxicity to normal cells [30]. The identification of antibodies targeting WGA in human serum indicates its translocation and interaction with the immune system [31]. Owing to the lack of in vivo studies, however, conclusions on the contribution of WGA to NCWS remain speculative [28,32,33].

Because WGA potentially contributes to symptoms of NCWS, this study aimed to determine the impact of sourdough fermentation on the fate WGA in wheat sourdoughs. The experimental design specifically assessed whether acidification and/or the reduction of disulfide bonds contribute to the degradation of WGA in wheat sourdoughs. The study was intended to be a first step in identifying whether sourdough fermentation reduces the content of WGA.

## 2. Materials and Methods

### 2.1. Sourdough Preparation

Two types of flour were tested, one pure wheat cultivar and one commercial whole wheat flour. The pure wheat cultivar was the heritage variety Red Fife. Wheat grains from the pure cultivar were ground using a 0.5 mm screen on a Retsch ZM200 ultra centrifugal mill (Retsch, Haan, Germany) and stored at −20 °C.

The strains used in these experiments were the homofermentative *Latilactobacillus sakei* TMW1.22, isogenic to LTH677 [34], the heterofermentative *Fructilactobacillus sanfranciscensis* DSM20451^T^, a sourdough isolate, and its isogenic derivative *F. sanfranciscensis* DSM20451Δ*gshR* which lacks glutathione reductase [19,35]. Chemically acidified dough was prepared by adding lactic acid (DL-lactic acid 90%, Sigma-Aldrich, Oakville, ON, Canada) and acetic acid (acetic acid glacial 99.7%, Sigma-Aldrich, Canada) at a 4 to 1 ratio to a pH of 4.

Strains were grown in modified De Man, Rogosa and Sharpe (mMRS), composed of the following components per litre: 10 g of maltose, 5 g of glucose, 5 g of fructose, 10 g of peptone, 5 g of yeast extract, 5 g of beef extract, 4 g of K_2_HPO_4_, 2.6 g of KH_2_PO_4_, 3 g of NH_4_Cl, 0.5 g of L-Cys HCl, 1 g of tween, 50 mg of MnSO_4_, 100 mg of MgSO_4_, 10 g of malt extract and 15 g of agar. The strains were cultivated at 30 °C under anaerobic conditions. Media used to cultivate *F. sanfranciscensis* DSM20451Δ*gshR* additionally contained 10 ppm of erythromycin. The identity of the strains was confirmed using polymerase chain reaction (PCR) as described [19].

Sourdoughs were prepared by mixing whole wheat flour and sterile tap water including the culture in a ratio 1:1 with a sterile wooden tongue depressor. Sourdoughs were prepared with one of the three strains, or by chemical acidification, and incubated at 30 °C for 24 h. Samples were taken after mixing and after 24 h to determine the pH, cell counts and the content of WGA. Samples for WGA analysis were stored at −80 °C. Three independent biological replicates were prepared per sample.

To determine the impact of proteases on the WGA content, protease from *Aspergillus oryzae* (Sigma Aldrich, also marketed as Flavorzyme by Novozyme) was used in one set of sourdoughs. Fifteen grams of sourdough were mixed with 25 µL of protease solution with more than 500 amino peptidase units per gram and incubated for 24 h at 30 °C.

To determine pH of the sourdoughs, 1 g of sourdough was mixed with 9 mL of sterile 18 mΩ water and the pH was measured with a pH meter. Cell counts of sourdoughs were determined by diluting 1 g of sourdough with 9 mL of sterile 18 mΩ water, followed by serial 10-fold dilutions with sterile peptone saline solution consisting of 10 g of peptone and 9 g NaCl per liter. The identity of the bacteria that dominated the fermentation was confirmed by comparing colony morphology from initial cell count plates with the morphology from the plates after fermentation.

### 2.2. WGA Analysis by Size Exclusion High Performance Liquid Chromatography (SEC-HPLC)

WGA (Sigma Aldrich, Oakville, ON, Canada) was tagged with fluorescein isothiocyanate (FITC, Sigma-Aldrich) as described [36]. WGA (2 mg, 20 mg or 15 mg) was dissolved in 1.5 mL of 0.1M sodium carbonate. FITC was dissolved in 1 mL of dimethyl sulfoxide (DMSO) to a concentration of 1 mg, 10 mg or 1.5 mg/mL. FITC in DMSO was added to the WGA solution at a rate of 0.25 mL every 15 min and the FITC-WGA solution was incubated in the dark at 6 °C for 8 h with gentle agitation. NH_4_Cl was added to a final concentration of 50 mM to stop the reaction, and the reaction was incubated for 2 h in the dark at 6 °C with gentle agitation. PD-10 desalting columns (GE Healthcare, Mississauga, ON, Canada) were used to collect the WGA-FITC conjugate and to remove unreacted FITC and salts. The protein conjugate was stored at 4 °C in the dark. Sourdoughs were prepared with the addition of 250 µg WGA-FITC conjugate per g of sourdough.

WGA was extracted based on the protocol of Baieli et al. [23]. In short, 1 g of sourdough was thawed at room temperature, mixed with 8 mL of 0.05 M HCl and incubated overnight with 300 rpm agitation at 25 °C. The pH was adjusted to 7 with NaOH, samples were centrifuged at 1396 rcf for 15 min, and the supernatant was collected and stored at −80 °C. Prior to HPLC analysis, samples were filtered through a 0.2 μm nylon filter and separated on a Superdex 200 Increase column (GE Healthcare) with a separation range from 10 kDa to 600 kDa. The eluent contained 0.1 M sodium phosphate, 0.15 M NaCl and 0.05 M N-acetyl glucosamine at pH 7.0; the flow rate was 0.5 mL/min at a temperature of 25 °C. FITC-labeled WGA was detected with a fluorescence detector at an excitation wavelength of 488 nm and an emission wavelength of 530 nm. The column was calibrated with the following standards: bovine serum albumin (BSA, 67 kDa), β-lactoglobulin (35 kDa), lysozyme (14 kDa), vitamin B12 (1.3 kDa) and glutathione (0.3 kDa). A flour sample without WGA-FITC conjugate was also tested as a blank. Blanks and standards were detected with a UV detector at 210 nm.

### 2.3. WGA Quantification with Enzyme-Linked Immunosorbent Assay (ELISA)

WGA was extracted as indicated above. The WGA ELISA kit was obtained from MyBioSource (San Diego, CA, USA) and tests were performed following the supplier’s instructions. Briefly, samples were thawed and diluted 100 times with deionized water to obtain WGA concentrations between 0.25 µg WGA/L to 8 µg WGA/L. Each sample was tested in technical repeats and a standard curve was prepared on every day an ELISA was performed. The sample diluent provided by the kit served as reagent blank. After addition of samples (50 µL), 100 µL of HRP-conjugate were added and the plates were incubated for 60 min at 37 °C. After incubation, the wells were washed four times with washing solution and 50 µL each of chromogen solutions A and B were added. The plates were incubated in the dark at 37 °C for 15 min, 50 µL of stop solution were added and the absorbance at 450 nm was determined.

To establish the possible effect of a reducing vs. an oxidative environment on the ELISA detection method, a test with Red Fife flour was performed. Three different samples were prepared: (1) 0.5 g flour + 0.5 mL sterile tap water, (2) 0.5 g flour + 0.5 mL of 5% H_2_O_2_, and (3) 0.5 g flour + 0.5 mL of 0.01 M glutathione (GSH). After mixing samples were collected and stored at −80 °C to analyze the WGA content as described above.

### 2.4. Statistical Analysis

The data was analyzed with two way ANOVA using the Shapiro-Wilk normality test and the Holm-Sidak post hoc analysis. The data obtained using ELISAs and the heritage wheat cultivar Red Fife was analyzed using one way ANOVA analysis, comparing the samples against each other and against the flour control.

## 3. Results

### 3.1. Microbial Growth and Metabolism in Wheat Sourdoughs

Cell counts and the pH of doughs were monitored to verify microbial growth and metabolism, and to confirm the identity of the fermentation organisms with the inoculum (Table 1). Growth and acidification of sourdoughs by *F. sanfranciscensis* and *L. sakei* matched prior observations. In addition, a uniform colony morphology that matched the respective inocula was observed on all plates, demonstrating that the strains used as inoculum accounted for more than 99% of bacterial cells in the respective sourdoughs (data now shown).

### 3.2. WGA Analysis Using Size Exclusion Chromatography (SEC)-HPLC

**Figure 1:**
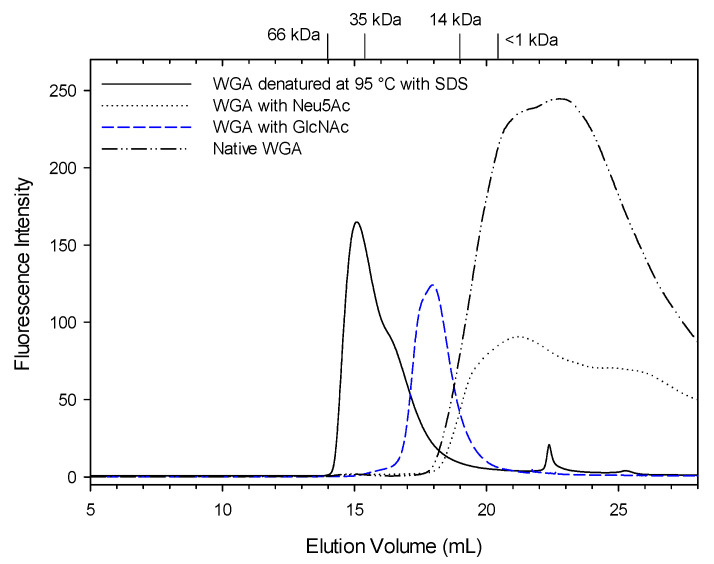
Wheat germ agglutinin **(**WGA) under four different conditions: Native WGA, WGA with N-acetyl neuraminic acid, WGA with N-acetyl glucosamine and WGA after heating with SDS buffer at 95 °C for 7 min. Arrows indicate the molecular weight of standards eluting at that volume. The standards used to determine molecular size were: bovine serum albumin (BSA) 66 kDa, β-lactoglobulin 35 kDa, lysozyme 14 kDa, vitamin B12 1.3 kDa and glutathione 0.3 kDa.

WGA was analyzed using SEC-HPLC after fermentation of sourdough with addition of fluorescence-labeled WGA. Native WGA eluted much later than expected on the basis of its molecular weight (Figure 1), likely because the lectin domain of WGA interacts with the solid phase of the dextran column. Therefore, the HPLC eluent was modified by denaturing WGA at 95 °C in presence of mercapto-ethanol and SDS, or by adding N-acetyl neuraminic acid (Neu5Ac) or N-acetyl glucosamine (GlcNAc) as ligands for the lectin domain of WGA (Figure 1). Addition of GlcNAc shifted the elution volume of WGA, confirming that the elution of native WGA was retarded by interaction with the dextran matrix of the column; addition of Neu5Ac, however, had no effect (Figure 1). WGA eluted fastest after denaturation with heat, reducing agents and SDS; however, the peak at 15 mL corresponds to 35 kDa, the molecular weight of the WGA dimer, and a peak corresponding to the molecular weight of the WGA monomer at 17 mL elution volume was observed only as a peak shoulder (Figure 1). Subsequent experiments were carried out without denaturation but with HPLC eluent containing GlcNAc.

**Figure 2:**
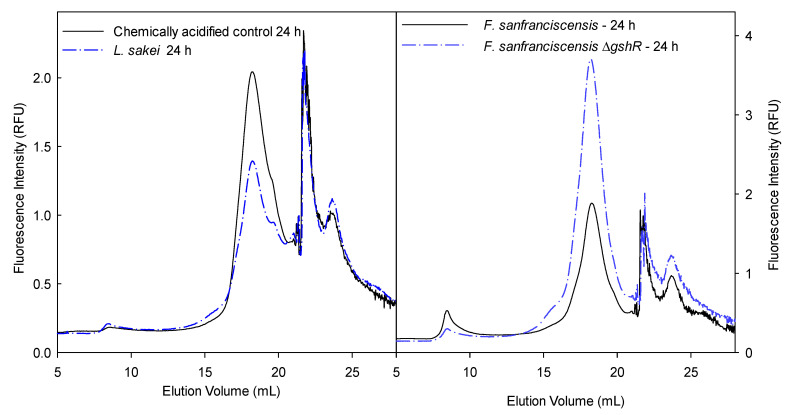
Chromatogram of fluorescence labeled WGA. WGA was extracted from chemically acidified doughs after 24 h of incubation and compared to *Latilactobacillus sakei* sourdough after 24 h of fermentation (left panel) and extracted from doughs fermented with *F. sanfranciscensis* DSM20451 Δ*gshR* and compared to *F. sanfranciscensis* DSM20451 after 24 h of fermentation (right panel).

Extracts from triplicate independent sourdough fermentations of commercial whole wheat flour with addition of the FITC-WGA conjugate were analyzed by HPLC-SEC. Replicate analyses were consistent with respect to the peak pattern but not with respect to the peak intensity (data not shown) and therefore support qualitative interpretation only. Chromatograms from the chemically acidified doughs and from doughs fermented with *L. sakei* show a major peak at 18 mL elution volume, corresponding to the WGA dimer; peaks eluting at higher elution volumes may indicate proteolytic degradation of WGA, or interaction of its lectin domain with the solid phase of the SEC column (Figure 2).

The chromatogram obtained with extracts of sourdoughs fermented with *F. sanfranciscensis* DSM20451 differed from all other sourdoughs including those fermented with *F. sanfranciscensis* DSM200451Δ*gshR*, as a peak was observed at an elution volume of 8 mL, corresponding to the void volume of the column (Figure 2). This peak corresponds to high molecular weight proteins. The peak was absent in *F. sanfranciscensis* DSM200451Δ*gshR*, suggesting that glutathione-mediated thiol exchange reactions resulted in incorporation of FITC-labeled WGA in the gluten macropolymer. This high molecular weight fluorescent peak was also observed in samples that were extracted after mixing, corresponding to a fermentation time of 15–30 min (data not shown). Dough mixing and bulk proof support thiol exchange reactions but do not provide sufficient time for proteolytic activity, further supporting the interpretation that thiol-exchange reactions are decisive for the fate of WGA in sourdough.

Overall, the interaction of the lectin domain of WGA with the dextran matrix of the SEC columns, and the variability of the fluorescence intensity of samples extracted from replicate sourdoughs confounded conclusions drawn from analysis of sourdoughs spiked with FITC-WGA conjugates. Nevertheless, the data provided preliminary evidence that the fate of WGA in sourdough is dependent on thiol exchange reactions that are catalyzed by glutathione. Subsequent analyses focused on ELISA detection of WGA.

### 3.3. WGA Quantification Using Enzyme Linked Immunosorbent Assay (ELISA)

**Figure 3:**
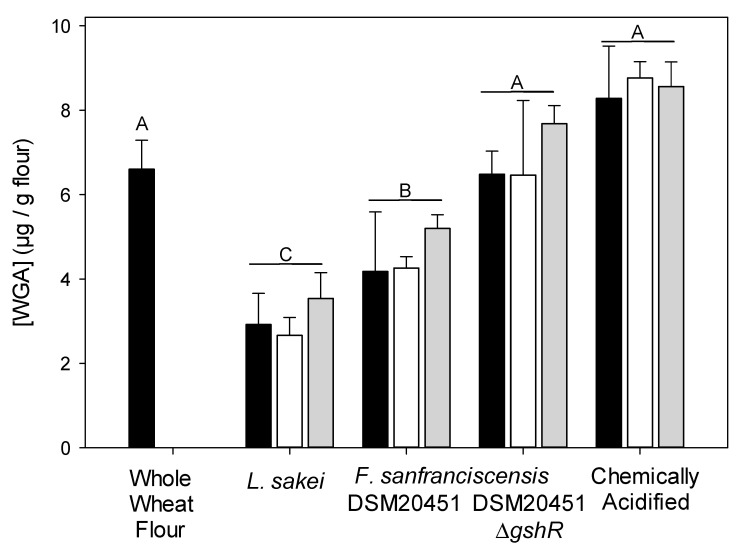
WGA content (μg WGA/g dry matter) of different sourdough samples (*n* = 3 biological replicates) taken after 0.5 h (black bars) or 24 h (white bars) of fermentation, or after 24 h of fermentation with addition of protease from *Aspergillus oryzae* at 30 °C (gray bars). Unfermented flour was used as control. Samples fermented with different strains or different conditions differ significantly (*p* < 0.001) unless they share a common capital letter. Samples fermented with the same strain for 0.5 h or for 24 h with or without addition of protease were not significantly different (*p* > 0.05).

WGA was quantified by ELISA after mixing and bulk proof, or after 24 h of fermentation (Figure 3). Consistent with the SEC analysis, samples obtained after mixing did not differ from samples after 24 h of fermentation (*p* > 0.05) (Figure 3). The WGA content was lowest in sourdoughs fermented with *F. sanfranciscensis* DSM20451 and *L. sakei* TMW 1.22 (Figure 3). The WGA content of sourdoughs fermented with *F. sanfranciscensis* DSM20451 and DSM20451Δ*gshR* differed significantly (*p* < 0.001), demonstrating that the reduction of oxidized glutathione by the glutathione reductase activity of *F. sanfranciscensis* reduced the WGA content of sourdoughs.

The addition of protease resulted in a more liquid consistency of the doughs (data not shown), indicating degradation of gluten proteins, but did not reduce the WGA content of sourdoughs (*p* > 0.05).

To verify whether oxidizing or reducing conditions affect WGA levels in dough, doughs prepared with flour from Red Fife but without addition of bacteria were analyzed by ELISA (Table 2). The addition of the oxidizing or reducing chemicals, hydrogen peroxide and glutathione, respectively, did not change the WGA content of doughs (*p* > 0.05) (Table 2), suggesting that biological reduction of oxidized glutathione acts differently from chemical oxidation or reduction of flour thiols.

**Figure 4:**
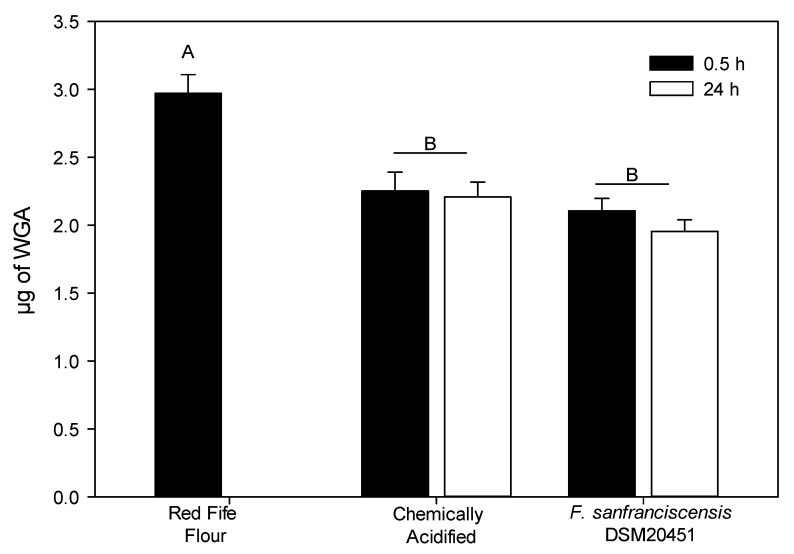
WGA content (μg WGA/g flour) of sourdough prepared with Red Fife pure cultivar flour (*n* = 3) after 24 h of fermentation with *F. sanfranciscensis* DSM20451. Chemically acidified doughs and Red Fife flours were used as controls. Samples fermented with different strains or different conditions differ significantly (*p* < 0.001) unless they share a common capital letter. Samples fermented with the same strain for 0.5 h or for 24 h were not significantly different (*p* > 0.05).

Fermentations were also performed using flour from pure wheat cultivar Red Fife. In these experiments, the strain *F. sanfranciscensis* DSM20451 also reduced the WGA content compared to the flour control. When using pure cultivar Red Fife flour, the WGA content of chemically acidified doughs was comparable to sourdough fermented with *F. sanfranciscensis* DSM20451^T^ (Figure 4).

## 4. Discussion

This study aimed to determine the fate of WGA and to assess the contribution of thiol-exchange reactions and of proteolysis to WGA modifications during sourdough fermentation. The role of thiol-exchange reactions was assessed by comparing *F. sanfranciscensis* DSM20451 with its isogenic glutathione-reductase negative mutant *F. sanfranciscensis* DSM20451Δ*gshR* [19,20]; the role of proteolytic activity was assessed by protease addition to sourdoughs, and by the use of chemically acidified controls [12]. This study analysed sourdoughs that were fermented with defined strains of lactic acid bacteria. This approach allows an assessment of the contribution of specific metabolic traits—acidification, glutathione reductase activity—to the degradation of WGA; however, sourdoughs used in artisanal and industrial practice typically include several species of lactic acid bacteria and additionally include sourdough yeasts or baker’s yeast [37]. Consortia of lactobacilli and sourdough yeasts were reported to degrade ATI more efficiently than pure cultures of lactobacilli [18] and thus may also impact WGA degradation.

### 4.1. Proteolysis in Sourdough and Contribution to WGA Degradation

*L. sakei* and *F. sanfranciscensis* do not express extracellular proteases. The genome of *F. sanfranciscensis* DSM20451 does not encode extracellular proteases [38,39], the strain does not exhibit proteolytic activity [38] and does not grow in sourdough if wheat aspartic proteases are inhibited by protease inhibitors [38]. Neither the genome of *L. sakei* 23K nor the genome of the type strain of the species encode for extracellular proteinases [39,40], and protein BLAST analysis with PrtP of *Lactobacillus helveticus* as query sequence confirmed that none of 54 *L. sakei* genomes that are currently available encode for an extracellular protease. Cell lysis releases intracellular peptidases but the effect of cell lysis depends on the type of sourdough and the fermentation time. The release of intracellular peptidases by autolysis is desirable in long-ripened cheeses [41], but is less likely to make a major contribution in sourdoughs where cell lysis is less extensive because type I sourdoughs are fermented for less than 24 h [12].

Proteolysis of highly disulfide bonded proteins including gluten and ovo-transferrin from egg white is dependent on the disruption of the quaternary and tertiary structure of the protein by disruption of disulfide bonds [20,42]. *F. sanfranciscensis* increases free thiol levels in sourdough through glutathione reductase activity [19,35,43] while free thiol levels decrease in sourdoughs fermented with *L. sakei, Schleiferilactobacillus perolens* or in chemically acidified doughs. Other heterofermentative lactobacilli accumulate thiols with different enzyme systems [19] and heterofermentative lactic acid bacteria rapidly reduce the redox potential of sourdough in a strain specific manner [11,44].

### 4.2. Fate of WGA during Sourdough Fermentation

Information on degradation of WGA during sourdough fermentation has to date not been available; prior reports on the effects of sourdough on nutritional characteristics of wheat germ flour did not consider WGA as it was assumed that its biological activity is eliminated during baking [45]. The concentration of WGA in white wheat flour is low, approximately 4 µg/g of flour, while whole wheat flour has a higher content of around 30 µg/g of flour [6]. This study assessed the fate of WGA by following the degradation of FITC-labeled WGA in sourdough fermentations, and by quantification of WGA through ELISA. The use of FITC-labeled proteins was previously used to determine the degradation of gluten proteins [46] and, more recently, dissociation and degradation of the ATI [18], but the results were interpreted qualitatively rather than quantitatively because the variability of extraction and quantification of fluorescent-labeled proteins adds to the variability of proteolytic activity among replicate sourdough fermentations [17,42,47]. In addition, the analysis of the size distribution of FITC-labeled WGA or its hydrolysis products was confounded by the interaction of the protein with the stationary phase of the column.

ELISA quantification of WGA demonstrated that sourdough fermentation reduces the concentration of WGA in whole wheat doughs. SEC-HPLC analysis of sourdoughs with fluorescein-labeled WGA and ELISA quantification of WGA both demonstrated that the fate of WGA is dependent on glutathione reductase activity of *F. sanfranciscensis*. ELISA quantification documented a significant difference in the WGA content of sourdoughs fermented with *F. sanfranciscensis* and *F. sanfranciscensis ΔgshR*; SEC-HPLC analysis documented that fluorescein-labeled WGA was linked to high molecular weight proteins in sourdoughs fermented with the *F. sanfranciscensis* wild type strain but not in sourdoughs fermented with the glutathione reductase-deficient mutant. This finding conforms to the highly disulfide-cross-linked structure of WGA which includes 32 disulfide bonds per dimer [24]. Of note, fermentation with *L. sakei* also reduced the WGA content although this strain oxidizes thiols in sourdough, in contrast to *F. sanfranciscensis*, which increases the thiol content [35]. The reduction of the WGA content was not enhanced by protease addition, demonstrating that thiol metabolism and the impact of lactobacilli on the redox potential are the main mechanisms for WGA conversion. The comparison of the pure cultivar flour and commercial whole wheat flour suggests that the type of flour and/or flour conditioning at the mill also impacts the fate of WGA.

### 4.3. Stability of WGA in Wheat Baking

Whole wheat bread is less widely consumed than white bread; in Canada, 1909 products were produced with white wheat flour compared to only 330 products with whole wheat flour [48]. The thermal inflection points of WGA denaturation is 65 °C but reduction of more than 80% of the carbohydrate binding activity requires heating to 90 °C for more than 10 min [21]. SEC-HPLC analysis of WGA in the present study suggested that heating to 95 °C in presence of SDS and reducing agents reduced or eliminated carbohydrate binding of the lectin domain but did not change the homo-dimeric structure of the protein. Even partial reduction of WGA with dithiothreitol reduces the carbohydrate binding of the lectin domain and thus alters its biological activity [49]. In addition, the denaturation of WGA with SDS occurs only in presence of reducing agents that disrupt its disulfide bonds [50,51] and thus the reduction of disulfide bonds of WGA in sourdough fermentation may impact the thermal stability of WGA.

## 5. Conclusions

Sourdough fermentation improves nutritional properties of wheat bread by removal of anti-nutritive factors and by altering the digestibility of macronutrients [52,53]. A potential role of sourdough fermentation to improve the tolerance towards wheat bread of individuals with NCWS is suggested by multiple and consistent reports on (social) media but, to date, supported by only a few studies on the fate of wheat components that are implicated in NCWS [9,10,18,54] and even fewer clinical studies [55,56]. Sourdough fermentation reduces FODMAP levels of wheat and rye bread. The extent depends on the fermentation organism and fermentation conditions [10,54] and reduced FODMAP levels did not consistently result in improved tolerance by individuals with NCWS [55,56]. Recent reports also suggest that the concentration of amylose trypsin inhibitor of wheat is reduced in wheat sourdough bread [18]. Multiple wheat components contribute to NCWS and different individuals respond to different stimulants [4,5,6,7].

The present study documents that fermentation with homofermentative and heterofermentative lactobacilli reduces the content of WGA, a potential contributor to NCWS, in wheat sourdoughs, and the reduction of WGA is dependent on thiol-metabolism and redox potential but not on proteolysis. Prior reports on fermentation of wheat germ assumed that WGA is denatured during baking [45]; however, comparison of the thermal stability of WGA [21] with the crumb temperature during baking [57] suggests that WGA may not be fully denatured in all products. The impact of WGA on human health and specifically to NCWS remains controversial because adverse effects were observed in vitro [27,28,29], but suitable in vivo studies remain elusive [33,58]. Therefore, the reduction of the WGA content by sourdough fermentation reported in the present study is likely to add one piece to the large but rather incomplete puzzle that relates wheat components and sourdough fermentation to the (in)tolerance of wheat products in NCWS and irritable bowel syndrome.

## Figures and Tables

**Table 1:** pH and cell count of sourdoughs with commercial whole wheat flour and Red Fife wheat flour (*n* = 3 biological replicates).

	pH			Cell Counts		
Strain	0.5 h	24 h	24 h + Protease	0.5 h	24 h	24 h + Protease
**Commercial Whole Wheat Flour**						
*Lt. sakei* TMW1.22	5.78 ± 0.12	4.00 ± 0.03	3.99 ± 0.03	1.0 ± 0.1 × 10^8^	1.1 ± 0.2 × 10^9^	1.3 ± 0.2 × 10^9^
*F. sanfranciscensis* DSM20451	5.85 ± 0.1	4.14 ± 0.02	4.22 ± 0.01	7.3 ± 0.7 × 10^7^	1.2 ± 0.8 × 10^9^	1.2 ± 0.8 × 10^9^
*F. sanfranciscensis* DSM20451 Δ*gshR*	5.75 ± 0.05	3.99 ± 0.17	4.06 ± 0.19	1.7 ± 0.8 × 10^7^	1.8 ± 1.3 × 10^9^	2.1 ± 1.6 × 10^9^
Chem. acidified	4.04 ± 0.02	4.04 ± 0.03	4.09 ± 0.01	n.d. *	n.d.*	n.d. *
**Red Fife Wheat Flour**						
*F. sanfranciscensis* DSM20451	6.01 ± 0.01	3.84 ± 0.01	n.d. *	9.5 ± 5.3 × 10^7^	1.1 ± 0.7 × 10^9^	n.d. *
Chem. acidified	4.07 ± 0.11	3.93 ± 0.03	n.d. *	n.d. *	n.d. *	n.d. *

* n.d. = Not determined.

**Table 2:** WGA content of doughs prepared with whole wheat flour prepared with the wheat cultivar Red Fife.

	(WGA) (μg WGA/g Flour)
Glutathione	3.03 ± 0.03
Hydrogen Peroxide	3.05 ± 0.61
Water	2.97 ± 0.14

## Data Availability

The datasets generated for this study are available on request to the corresponding author.
